# Integrative meta-analysis of the OSCC microbiome: from niche-specific heterogeneity to universal network and functional signatures

**DOI:** 10.1080/20002297.2026.2730638

**Published:** 2026-09-07

**Authors:** Hee Sam Na, Jung Hwa Park, Tae Sung Kim

**Affiliations:** a Department of Microbiology and Immunology, Chonnam National University Medical School, Hwasun, Jeollanam-do, Republic of Korea; b Department of Oral Microbiology, School of Dentistry, Dental and Life Science Institute, Pusan National University, Yangsan, Republic of Korea; c Education and Research Team for Life Science on Dentistry, Pusan National University, Yangsan, Republic of Korea

**Keywords:** Oral microbiome, oral squamous cell carcinoma, biomarker, integrative meta-analysis, microbial network

## Abstract

**Background:**

While the association between oral squamous cell carcinoma (OSCC) and microbial dysbiosis is well-established, high inter-study heterogeneity has historically hindered the identification of universal biomarkers.

**Objective:**

To explore the profound influence of anatomical ecological niches on microbial profiles while identifying shared oncogenic signatures across diverse populations.

**Design:**

An integrative meta-analysis of independent OSCC microbiome cohorts was conducted.

**Results:**

Our results demonstrate that microbial community composition is predominantly driven by the sampling site rather than the disease state alone, with tissue-derived samples forming a distinct ecological cluster. Despite this site-specific dominance, a consistent core of anaerobic pathogens-including *Fusobacterium*, *Capnocytophaga*, and *Treponema* was significantly enriched in OSCC across multiple datasets. Network-based analysis identified *Fusobacterium* as top driver of microbial community restructuring. Diagnostically, saliva-based models achieved superior accuracy compared to tissue and mucosal models, suggesting that saliva serves as an integrative reservoir for oral pathological changes. Functional profiling further revealed a significant functional convergence toward pathways essential for rapid bacterial proliferation and environmental adaptation, such as ribosome biogenesis, DNA replication, and flagellar assembly.

**Conclusions:**

These findings emphasize the necessity of standardizing sampling protocols and provide a robust scientific basis for non-invasive, saliva-based precision diagnostic strategies for OSCC.

## Introduction

Oral squamous cell carcinoma (OSCC) represents a significant global public health challenge, accounting for the vast majority of oral malignancies and characterised by high morbidity and mortality rates [1–3]. Traditional risk factors such as tobacco use, alcohol consumption, and betel quid chewing are well-established contributors to OSCC development. However, emerging evidence suggests that the oral microbiome is a critical mediator in the initiation and progression of OSCC [4–7]. The oral cavity harbours a complex microbial ecosystem where dysbiosis can trigger chronic inflammation and the production of oncogenic metabolites, facilitating carcinogenesis through the activation of host signalling pathways [8].

Despite the proliferation of studies, identifying replicable microbial biomarkers across diverse populations remains difficult due to significant inter-study heterogeneity [4,9,10]. Current trends in OSCC microbiome research have largely focused on comparing the relative abundance of specific taxa between healthy controls and cancer patients. However, these abundance-centric approaches often yield inconsistent results a phenomenon frequently attributed to ‘batch effects’ arising from different geographical locations, dietary habits, and sequencing methodologies [4,6,7,9]. Furthermore, the oral cavity presents a unique challenge: the sampling site effect. Unlike the gut, where stool is a relatively uniform medium, oral microbial communities are highly structured by their anatomical niche [9,11,12].

These challenges also highlight the limitations of relying solely on differential abundance analyses to characterise the OSCC microbiome. In particular, most existing studies have largely overlooked microbial interaction networks. Microbes do not function in isolation their impact on the host is a result of complex co-occurrence patterns and community-level restructuring [13–15]. While a few studies in other fields, such as periodontitis and Parkinson's disease, have begun to explore network robustness [16–18], such analyses remain scarce in OSCC research. Traditional differential analysis methods like Linear Discriminant Analysis Effect Size (LEfSe) are limited in capturing these structural transitions. Therefore, there is a need to move beyond taxonomic diversity toward identifying keystone ‘drivers’ that orchestrate the transition from a healthy oral ecosystem to a pro-tumorigenic state.

To address these challenges, we employed a novel network-based integration framework called NetMoss. Unlike conventional abundance-based techniques, NetMoss identifies biomarkers by quantifying their contribution to network rewiring and is specifically designed to eliminate batch effects when integrating large-scale, multi-cohort data [19]. This method has recently proven effective in uncovering driver taxa in subgingival plaque during periodontitis [20] and identifying robust biomarkers for Parkinson’s disease by focusing on shifts in network modules rather than individual abundances [16].

In this study, we performed an integrative meta-analysis of 13 independent OSCC datasets, incorporating a wide range of sampling modalities including tissue, saliva, and mucosa. By applying the NetMoss framework, we aimed to (1) quantify the influence of sampling site effects across diverse cohorts and decouple niche-specific noise from disease-associated signals; (2) identify universal driver taxa, such as *Fusobacterium*, that consistently govern the restructuring of the OSCC-associated microbial network; (3) evaluate the diagnostic potential of these markers across different sampling sites, with a particular focus on the accuracy of saliva-based diagnostic; and (4) elucidate the functional convergence of the OSCC microbiota through metabolic profiling to identify conserved pathways supporting the tumour microenvironment. This integrative approach provides a high-resolution map of the OSCC microbiome, offering a robust scientific foundation for standardised non-invasive diagnostic strategies.

## Results

### Microbial diversity and community structure across OSCC datasets

**Figure 1.**
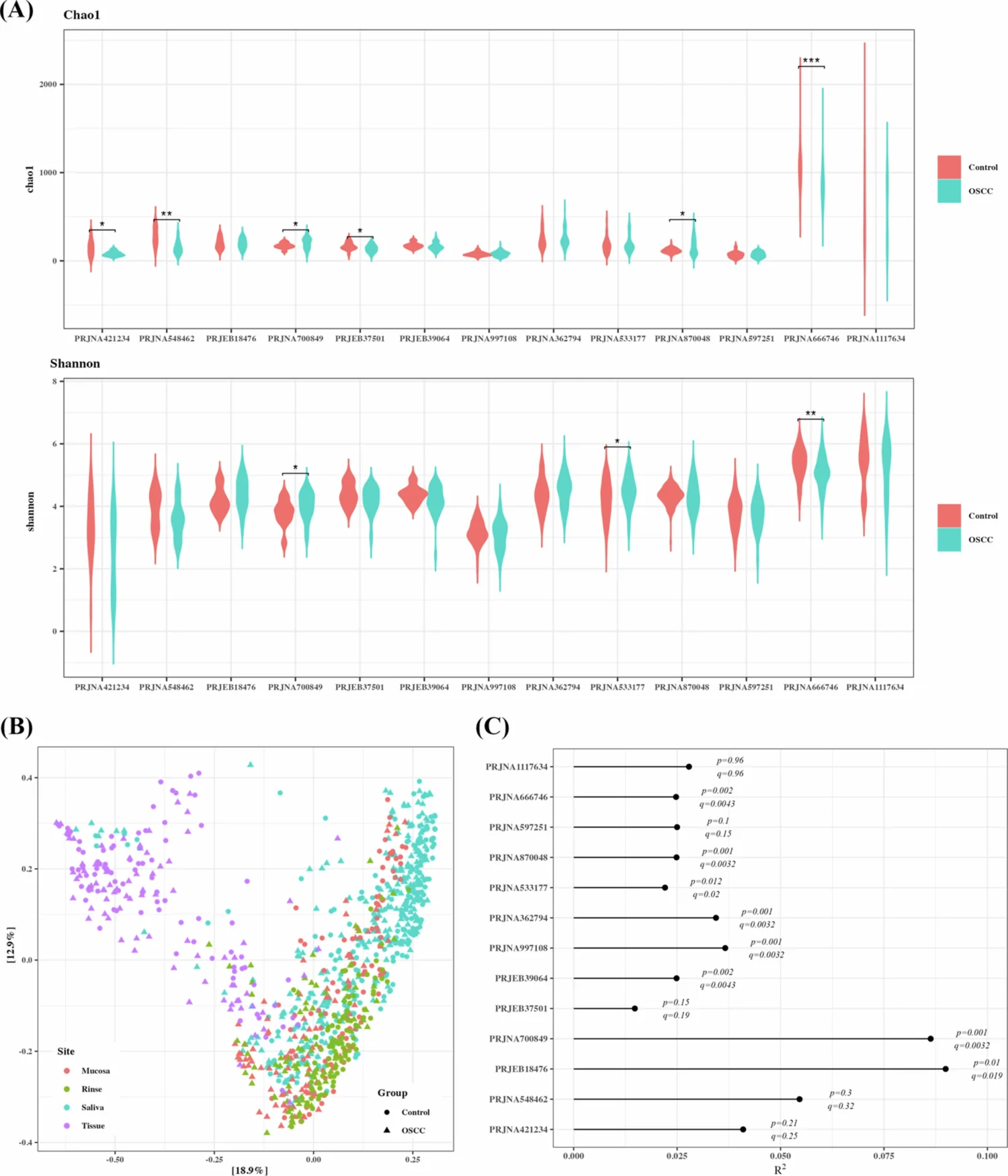
Microbial diversity across OSCC microbiome datasets. (**A**) Alpha diversity. Chao1 and Shannon indices were calculated for each dataset. Adjusted *p*-values are indicated as follows: *p < 0.05, **p < 0.01, ***p < 0.001. (**B**) Beta diversity. Principal coordinates analysis (PCoA) based on Bray–Curtis dissimilarity calculated from merged species-level relative abundance profiles. (**C**) Study-wise PERMANOVA results based on Bray-Curtis distances testing the effect of group within each study. Dots indicate the R^2^ values for each study, and the corresponding nominal *p* values and Benjamini-Hochberg-adjusted q values are shown alongside each point.

A total of 13 independent OSCC microbiome datasets representing saliva, mucosal, oral rinse, and tissue samples were included in this study (Table 1). Alpha diversity analysis revealed that the impact of OSCC on microbial richness and evenness varies significantly across studies (Figure 1A). Based on the Chao1 index, significant differences in microbial richness between Control and OSCC groups were observed in 6 out of 13 datasets (PRJNA421234, PRJNA548462, PRJNA700849, PRJEB37501, PRJNA870048, and PRJNA666746). However, the Shannon index, which integrates both richness and evenness, showed significant differences in only 3 datasets (PRJNA700849, PRJNA533177, and PRJNA666746). These findings indicate that while OSCC frequently alters microbial richness, its effect on the overall community structure is not universally consistent across different study populations.

**Table 1.** Summary of OSCC oral microbiome datasets included in the integrative analysis.

BioProject	Sampling site	Sample size (*n*)		16S target	Country	References
		Control	OSCC			
PRJNA421234	Saliva	16	14	V3-V4	New Zealand	Vesty et al. [21]
PRJNA548462	Saliva	10	10	V3-V4	India	Oyeyemi et al. [22]
PRJEB18476	Saliva	10	11	V4	Austria	Wolf et al. [23]
PRJNA700849	Saliva	8	16	V4	Brazil	Granato et al. [24]
PRJEB37501	Saliva	28	45	V3-V4	Taiwan	Chen et al. [25]
PRJEB39064	Saliva	27	27	V3-V4	Taiwan	Chen et al. [26]
PRJNA997108	Saliva	101	99	V4	Finland	Mäkinen et al. [27]
PRJNA362794	Mucosal	40	40	V3-V4	China	Zhao et al. [28]
PRJNA533177	Mucosal	50	50	V3-V4	China	Zhang et al. [29]
PRJNA870048	Oral rinse	91	91	V3-V4	Hong Kong	Zhu et al. [30]
PRJNA597251	Tissue	24	24	V3-V4	China	Zhou et al. [31]
PRJNA666746	Tissue	50	50	V3-V4	India	Sarkar et al. [32]
PRJNA1117634	Tissue	13	13	V3-V4	India	Not reported

Beta diversity was assessed using Principal Coordinates Analysis (PCoA) to visualise the similarity between microbial communities (Figure 1B). The results demonstrated that the sampling site (Tissue, Saliva, Mucosa, or Rinse) is the primary factor driving microbial composition, rather than the disease state. Specifically, tissue samples formed a distinct cluster in the PCoA plot, clearly separated from the other sampling modalities. Within each site, samples from the Control and OSCC groups exhibited considerable overlap, suggesting that the inherent microbial signature of the anatomical site remains dominant over disease-induced shifts.

To quantify the effect size of the disease on microbial variation and compare community structure within each study, PERMANOVA analysis of Bray-Curtis dissimilarities was performed for each of the 13 cohorts (Figure 1C). The effect size (R^2^) of OSCC status was generally low, ranging from approximately 0.02 to 0.09 across datasets. Despite these relatively modest effects, study-specific analysis demonstrated significant differences in community structure between Control and OSCC samples in the majority of cohorts. Specifically, after applying Benjamini-Hochberg multiple testing corrections, 8 of the 13 datasets remained statistically significant (q < 0.05), showing clear separation between groups: PRJNA666746, PRJNA870048, PRJNA533177, PRJNA362794, PRJNA997108, PRJEB39064, PRJNA700849, and PRJEB18476. In contrast, the remaining 5 datasets (PRJNA1117634, PRJNA597251, PRJEB37501, PRJNA548462, and PRJNA421234) showed no significant association between disease status and microbial composition.

These findings suggest that OSCC-associated shifts in overall microbial composition are present across multiple datasets, but their strength is study-dependent. Although larger cohorts more often showed significant Control-OSCC separation, this pattern was not consistent across all datasets, suggesting that factors beyond sample size and sampling site contributed to between-group differences.

### Taxonomic composition and enrichment patterns of the oral microbiota in OSCC

**Figure 2.**
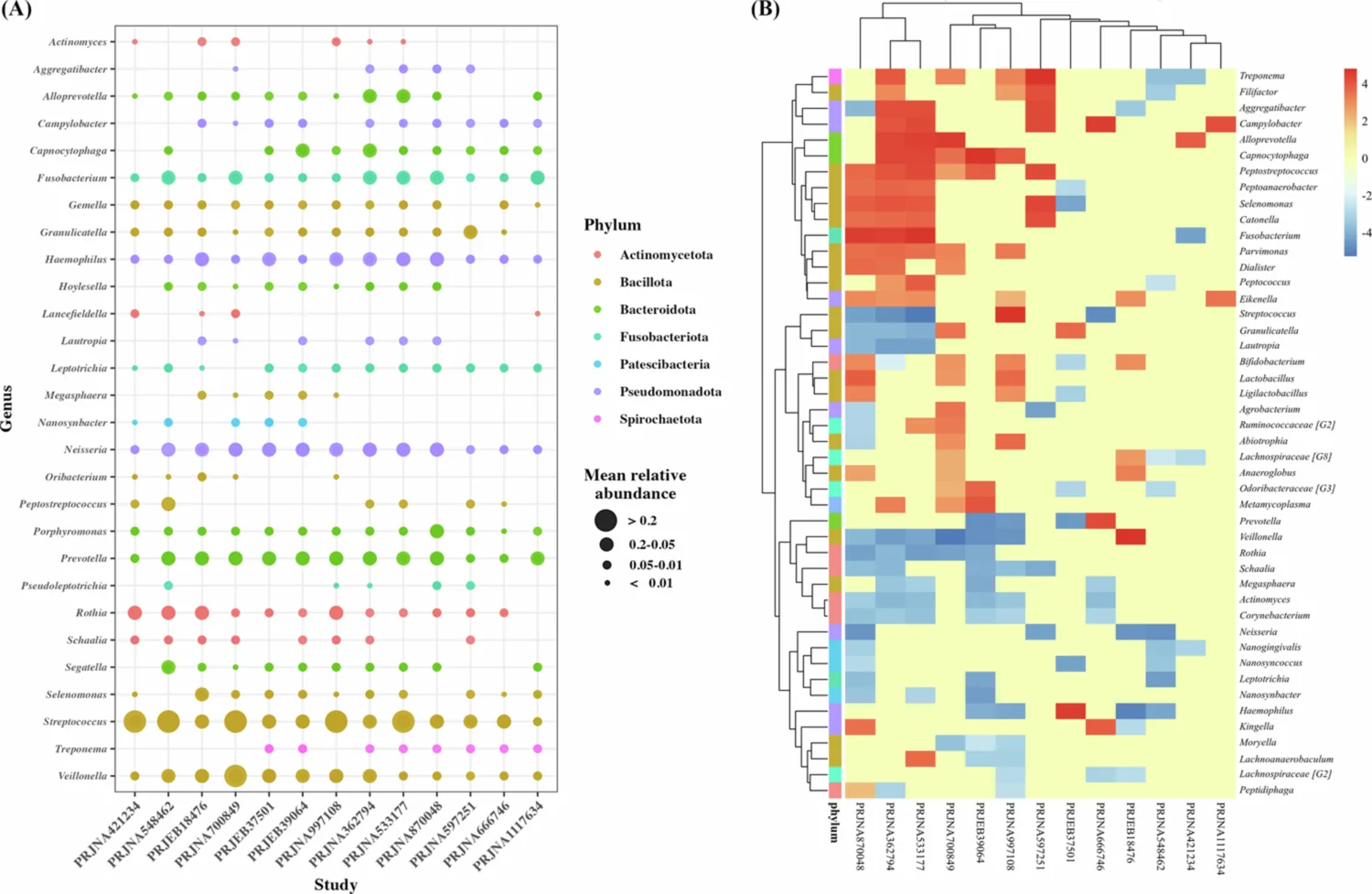
Study-consistent dominant genera and differentially abundant genus-level taxa across Control and OSCC cohorts. **(A)** Bubble plot showing shared dominant genera across studies. Genera were selected from the top 20 taxa ranked by mean relative abundance within each study and group, and only genera present in at least 4 studies were displayed. Bubble size represents categorised mean relative abundance, and bubble colour indicates phylum affiliation. **(B)** Heatmap of genus-level biomarkers identified by LEfSe in individual studies. Only significant genera observed in at least 3 studies were included. Signed LDA scores are shown, with positive scores representing OSCC-enriched taxa and negative scores representing Control-enriched taxa. Row annotation indicates phylum.

To identify the microbial signatures associated OSCC, the taxonomic distribution of the 13 datasets was analysed at the genus level. Relative abundance patterns of major genera across the datasets revealed both shared core taxa and distinct site-dependent differences (Figure 2A).

Across all studies, a consistent set of core taxa was observed at high mean relative abundance, including *Streptococcus*, *Veillonella*, *Prevotella*, *Haemophilus*, *Neisseria*, and *Fusobacterium*. Despite these commonalities, sampling site-specific patterns were evident. Genera such as *Oribacterium*, *Nanosynbacter*, and *Megasphaera* were predominantly detected in saliva-based datasets, while remaining rare or absent in mucosal and tissue-derived samples. Conversely, *Aggregatibacter* and *Alloprevotella* exhibited relatively higher abundance in mucosal datasets. Notably, tissue-derived datasets showed a marked reduction in several commonly observed genera, such as *Neisseria*, *Veillonella*, *Lautropia*, and *Hoylesella* compared to other sampling modalities.

The enrichment patterns of specific taxa were further explored using a heatmap of signed LDA scores, which revealed both study clustering and disease-associated trends (Figure 2B). Hierarchical clustering of the datasets showed that samples partially clustered according to their sampling site, with mucosal datasets and a subset of saliva datasets exhibiting similar microbial profiles. Consistent OSCC-associated enrichment was observed across multiple independent cohorts. Taxa significantly enriched in the OSCC group included genera such as *Fusobacterium*, *Capnocytophaga*, *Campylobacter*, *Alloprevotella*, *Treponema*, and *Filifactor* and *Peptostreptococcus*. According to the phylum-level annotation, these enriched taxa were predominantly associated with the phyla Fusobacteriota, Bacteroidota, and Spirochaetota (Figure 2B). In contrast, the control-enriched taxa were characterised by genera such as *Veillonella*, *Rothia*, *Actinomyces*, and *Corynebacterium*. These findings highlight that while a core oral microbiome exists, OSCC is associated with a shift toward a specific group of anaerobic and opportunistic pathogens across different study populations.

### Network-based identification of driver taxa and diagnostic performance across OSCC datasets

**Figure 3.**
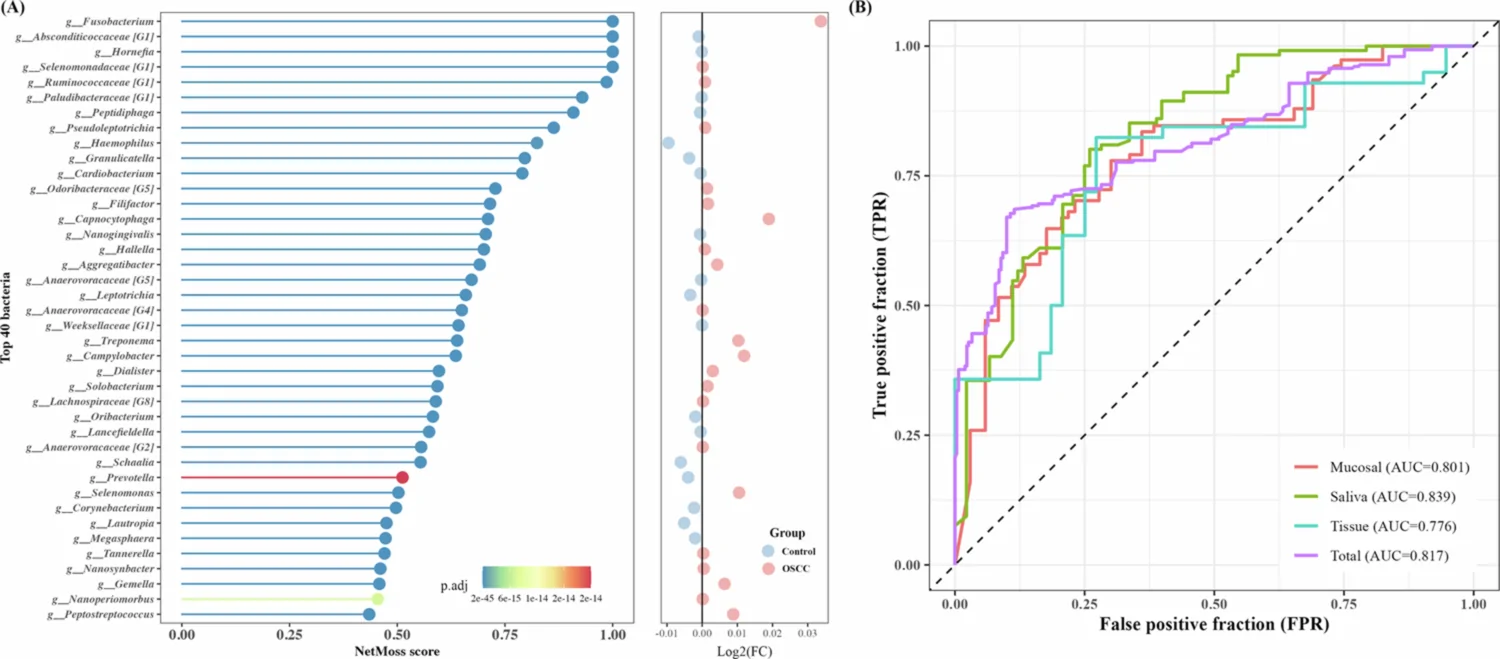
Network-based microbial biomarkers identified by NetMoss2 and their diagnostic performance in OSCC. (**A**) Top 40 genus-level taxa ranked by NetMoss score. The lollipop plot summarises the magnitude of the NetMoss score for each taxon, with colour indicating the adjusted *p* value. The accompanying plot displays log2 fold change between OSCC and Control, with positive values indicating OSCC enrichment and negative values indicating Control enrichment. (**B**) ROC curves showing the performance of NetMoss2-based classifiers for mucosal, saliva, tissue, and pooled samples. AUC values are provided for each curve.

To identify the key microbial taxa contributing to the structural differences between the Control and OSCC groups, we applied the NETMoss framework across the 13 datasets. NETMoss scores were calculated at the genus level and visualised alongside log2 fold change (FC) values to determine both the network-level importance and the enrichment patterns of specific taxa (Figure 3A).

Among the top 40 ranked bacteria, several genera exhibited high NETMoss scores, indicating a significant contribution to the microbial network restructuring associated with OSCC. Notably, *Fusobacterium* achieved the highest NETMoss score (1.0) and was consistently enriched in the OSCC group across the datasets, as indicated by its positive Log2(FC) value (Figure 3A). Other top-ranked taxa significantly enriched in OSCC included genera such as *Capnocytophaga*, *Treponema*, *Campylobacter*, *Gemella*, and *Peptostreptococcus*. Conversely, key network contributors enriched in the Control group included *Haemophilus*, *Schaalia*, and *Prevotella* (Figure 3A). Interestingly, certain taxa were identified as significant drivers due to their high NETMoss scores despite exhibiting relatively modest differences in relative abundance, highlighting the sensitivity of network-based analysis in capturing subtle but biologically relevant microbial shifts (Figure 3A).

To examine whether the pooled NetMoss findings were driven by individual datasets, we performed leave-one-dataset-out and leave-two-datasets-out sensitivity analyses (**Supplementary Figure S1 and S2**). A subset of the principal NetMoss markers was consistently retained across the omission scenarios, supporting the reproducibility of the main ecological signal. Nevertheless, the correlation between the complete-data NetMoss score ranking and the rankings obtained after dataset omission varied considerably, and several omission scenarios showed weak concordance. Thus, although several high-priority taxa remained detectable, the exact NetMoss scores and relative rankings were sensitive to study composition.

To evaluate the clinical utility of these network-derived microbial features, Receiver Operating Characteristic (ROC) curve analysis was performed across different sampling sites (Figure 3B). The microbial markers demonstrated robust discriminative performance, with Area Under the Curve (AUC) values exceeding 0.75 for all modalities. The saliva-based model achieved the highest diagnostic accuracy with an AUC of 0.839, followed by the integrated total dataset (AUC = 0.817) and the mucosal samples (AUC = 0.801) (Figure 3B). The tissue-based model showed a comparatively lower, yet still significant, performance with an AUC of 0.776 (Figure 3B). These results suggest that network-derived microbial signatures, particularly those obtained from non-invasive saliva samples, serve as highly effective biomarkers for distinguishing OSCC patients from healthy controls.

### Functional pathway profiles and shared metabolic signatures across OSCC datasets

**Figure 4.**
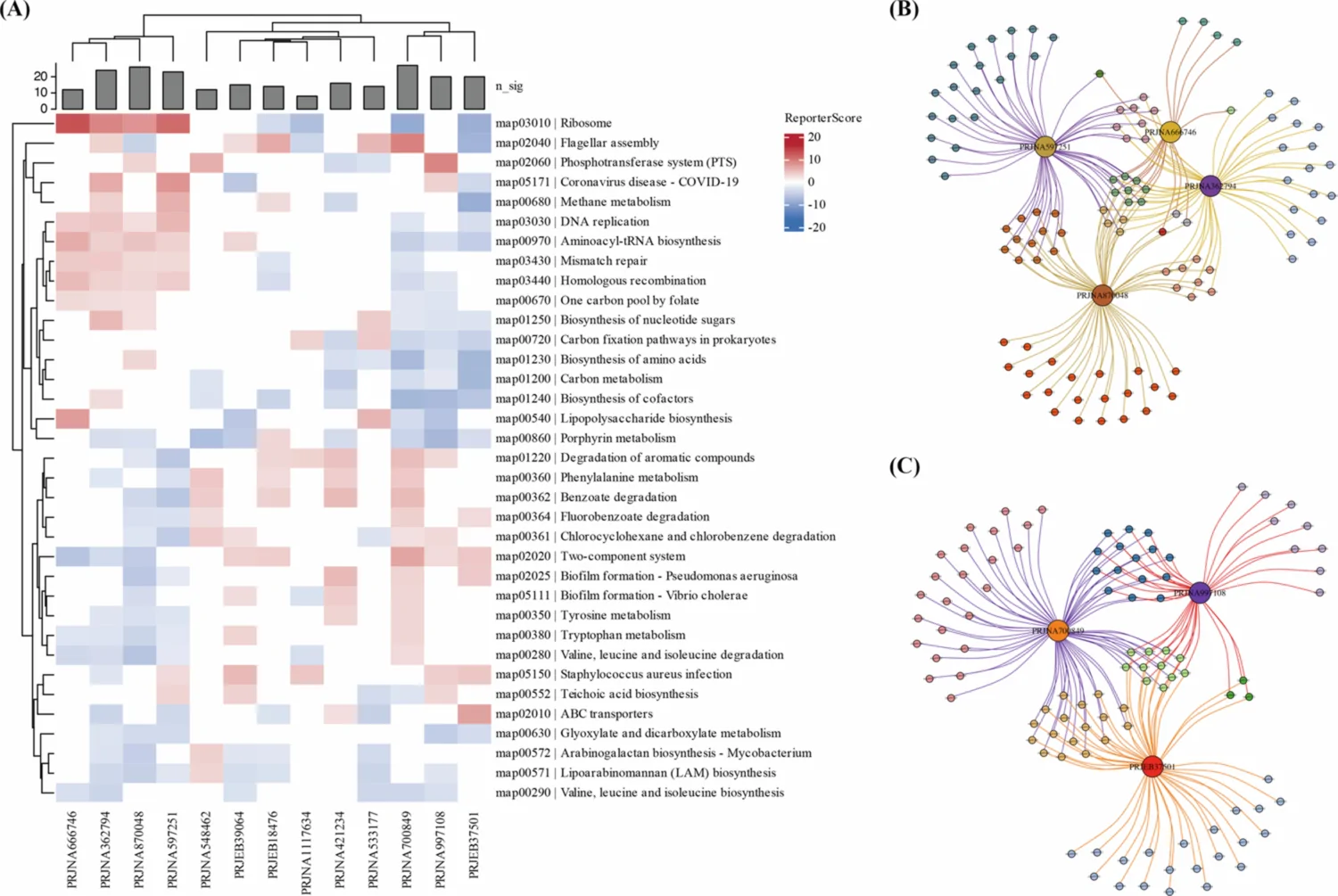
Cross-study functional pathway signatures inferred by PICRUSt. (**A**) Heatmap showing signed ReporterScore values for KEGG pathways across studies. Pathways detected as significant in at least 3 studies were retained, and studies with at least 5 significant pathways were displayed. Positive scores (red) indicate pathways enriched in OSCC, whereas negative scores (blue) indicate pathways enriched in Control. The bar plot above the heatmap (n_sig) indicates the number of significant pathways per study. (**B**) Network-style Venn diagram showing the overlap of significant pathways among PRJNA666746, PRJNA362794, PRJNA870048, and PRJNA597251. (**C**) Network-style Venn diagram showing the overlap of significant pathways among PRJNA700849, PRJNA997108, and PRJEB37501. In panels B-C, large labelled nodes represent studies, small nodes represent significant pathways, and connecting edges indicate pathway membership shared across studies.

To investigate the functional implications of the observed microbial shifts, we performed a metabolic pathway enrichment analysis based on the Kyoto Encyclopaedia of Genes and Genomes (KEGG) database across the 13 datasets. The functional profiles revealed heterogeneous patterns of pathway enrichment, with hierarchical clustering identifying three distinct study-specific groups based on their metabolic signatures (Figure 4A). Across a majority of the datasets, particularly in the first cluster (Cluster A), several pathways were significantly enriched in OSCC samples (positive ReporterScores, indicated in red). These included pathways critical for bacterial growth, genetic information processing, and motility, such as Ribosome (map03010), Flagellar assembly (map02040), Phosphotransferase system (PTS; map02060), Methane metabolism (map00680), DNA replication (map03030), Aminoacyl-tRNA biosynthesis (map00970), Mismatch repair (map03430), and Homologous recombination (map03440) (Figure 4A).

The consistent enrichment of these pathways suggests a functional shift toward increased bacterial proliferation and environmental adaptation within the tumour microenvironment. In contrast, core metabolic processes were predominantly enriched in the control samples (negative ReporterScores, indicated in blue) across several datasets, especially within the middle cluster of six studies (Cluster B). These pathways included carbon fixation pathways in prokaryotes (map00720), biosynthesis of amino acids (map01230), carbon metabolism (map01200), biosynthesis of cofactors (map01240), lipopolysaccharide (LPS) biosynthesis (map00540), and porphyrin metabolism (map00860) (Figure 4A).

Network analysis further highlighted subset-specific shared signatures among the datasets (Figure 4B and 4C). Cluster A, comprising PRJNA666746, PRJNA362794, PRJNA870048, and PRJNA597251, was characterised by a dense network of shared pathways highly enriched in OSCC samples (Figure 4B). Conversely, Cluster B, which included PRJNA700849, PRJNA997108, and PRJEB37501, exhibited a distinct functional profile, showing unique enrichment in pathways such as teichoic acid biosynthesis (map00552) within their respective Control groups (Figure 4A, 4C). These findings demonstrate that while taxonomic composition may vary, the oral microbiota in OSCC converge on common functional themes, particularly involving enhanced protein synthesis and DNA repair mechanisms.

## Discussion

Microbial dysbiosis within the tumour microenvironment has emerged as a critical factor influencing cancer initiation and progression, including in OSCC where oral microbiome alterations are closely associated with tumour development [7,33,34]. However, the high degree of inter-study heterogeneity compounded by the dominant influence of anatomical sites has limited the identification of reproducible biomarkers [12,35]. To better understand the microbial alterations associated with OSCC, integrative meta-analyses are needed to identify microbial signatures that are consistently associated with the disease. In this study, we integrated 13 independent OSCC cohorts to establish a high-resolution map of the cancer-associated microbiome, moving beyond simple abundance comparisons to network-based identification of key taxa. To further elucidate the biological impact of these microbial shifts, a metabolic pathway enrichment analysis based on the KEGG database was also performed.

Alpha diversity analysis showed that while OSCC frequently alters microbial richness, this effect is not universal significant differences in the Chao1 index were observed in only 6 out of 13 datasets, and the Shannon index which accounts for both richness and evenness showed significant shifts in only three cohorts. Similarly, while study-specific PERMANOVA of beta diversity identified significant differences in community structure between control and OSCC samples in the majority of cohorts (8 out of 13), this separation was notably absent in the remaining five datasets. This inconsistency suggests that factors beyond the disease state, such as varying sample sizes and technical batch effects, contribute to the baseline variability observed across OSCC microbiome research.

In addition, the anatomical sampling site exerts a more significant influence on microbial composition than the OSCC disease state itself. The PCoA analysis clearly demonstrates that tissue-derived samples cluster separately from saliva, mucosa, and rinse modalities, regardless of whether the samples were from the control or OSCC group. This ‘site effect’ is statistically supported by the relatively low PERMANOVA effect sizes (R [2] ranging from 0.02 to 0.09) for disease status across most datasets, suggesting that niche-specific signatures may mask subtle microbial shifts associated with malignancy [9,11,12]. Taxonomically, this niche-driven structure is reflected in the prevalence of specific genera like *Oribacterium* and *Nanosynbacter* in saliva, while mucosal datasets were characterised by higher abundances of *Aggregatibacter* and *Alloprevotella*. The marked reduction of common commensal genera such as *Neisseria* and *Veillonella* in tissue samples (specifically observed in PRJNA1117634) further highlights how the localised tumour environment selects for a specialised microbial niche. These findings are consistent with previous reports indicating that the oral cavity harbours highly distinct ecological niches (ex. keratinised vs non-keratinised mucosa), which likely contribute to baseline variability and may mask disease-associated shifts [35–37]. Consequently, identifying a universal OSCC signature requires accounting for these site-specific baselines to decouple localised ecological noise from microbial taxa consistently associated with OSCC.

Despite the high degree of heterogeneity between studies, our integrative meta-analysis identified a consistent ‘OSCC signature’ dominated by anaerobic and opportunistic pathogens. Using the NETMoss framework, *Fusobacterium* was identified as the top-ranked candidate network biomarker with a maximum score of 1.0, appearing consistently enriched in OSCC samples across multiple cohorts. Other highly ranked network-associated taxa included *Capnocytophaga*, *Treponema*, *Campylobacter*, and *Peptostreptococcus*. The identification of *Fusobacterium* as the highest-ranked network-association taxon is consistent with its known role in promoting oncogenesis. This is consistent with the established oncogenic role of *Fusobacterium nucleatum*, which has been shown to induce pro-inflammatory environments and activate signalling pathways, such as Wnt/β-catenin, that facilitate tumour growth and invasion [38–40]. Our findings suggest that *Fusobacterium* represents a consistently identified network-associated taxon across multiple OSCC cohorts.

The comparative analysis of diagnostic performance revealed that saliva-based microbial models achieved the highest accuracy (AUC = 0.839), outperforming tissue-based (AUC = 0.776) and mucosal-based (AUC = 0.801) models. This superiority of saliva as a diagnostic medium is likely because saliva acts as a comprehensive reservoir, collecting microbial signatures from various oral surfaces and the tumour site itself. This superiority of saliva as a diagnostic medium likely reflects its role as a comprehensive reservoir that integrates microbial signals from multiple oral surfaces, including the tumour site [41,42]. These results strongly support the development of saliva-derived microbial biomarkers as a highly effective, non-invasive screening tool for OSCC.

Functional enrichment analysis revealed that while taxonomic composition varies, the OSCC-associated microbiota converges on common metabolic signatures. OSCC samples across the majority of datasets (particularly Cluster A) showed significant enrichment in pathways related to protein synthesis (Ribosome), motility (Flagellar assembly), and DNA repair/replication (Mismatch repair, Homologous recombination). Conversely, control samples were enriched in core metabolic processes such as amino acid biosynthesis and carbon metabolism. The predicted enrichment of pathways related to genetic information processing and motility in OSCC-associated bacteria may reflect adaptation to the nutrient-rich yet competitive environment of the tumour, potentially contributing to cancer progression through the production of pro-inflammatory metabolites [10,33]. However, these findings are based on inferred functional profiles and require experimental validation.

Recent reviews have highlighted that inconsistent findings across OSCC microbiome studies are largely attributable to methodological and clinical heterogeneity, including differences in sampling procedures, sequencing platforms, analytical approaches, and patient characteristics, which have hindered the identification of reproducible microbial signatures [43,44]. Although accumulating evidence supports an association between the oral microbiome and OSCC, a causal relationship has not yet been firmly established [44]. Therefore, future studies should not only standardise sampling protocols but also carefully control methodological and host-related factors, such as sample collection techniques, patient characteristics, and recent antibiotic use, to improve the reproducibility and comparability of oral microbiome research.

Despite the strengths of this integrative analysis, this study has several limitations. First, important clinical variables, including smoking status, alcohol consumption, and periodontal status, could not be consistently considered across the included datasets because these data were not uniformly available. Second, although leave-one-dataset-out and leave-two-datasets-out analyses demonstrated that a subset of the key NetMoss markers was consistently retained, the exact NetMoss scores and relative ranking of individual taxa varied across omission scenarios, indicating that some datasets exerted a measurable influence on the pooled network structure. This variability likely reflects differences in population characteristics, sequencing protocols, taxonomic resolution, sample size, and other methodological factors among studies. Accordingly, larger meta-analyses with more consistently sampled cohorts and harmonised sequencing and analytical protocols will be necessary to establish more generalisable OSCC-associated microbial network markers.

In summary, this integrative meta-analysis demonstrates that while sampling sites dominate the oral microbial landscape, *Fusobacterium* was consistently identified as the highest-ranked network-associated taxon across the included datasets. The robust performance of saliva-based models provides a pathway for clinical application in early OSCC detection. Standardising sampling protocols remains a priority for future research to minimise site-specific noise and refine these global microbial signatures.

## Materials and methods

### Data collection and processing

Publicly available 16S rRNA sequencing studies on OSCC were identified through a literature search of PubMed using keywords related to OSCC, the oral microbiome, and 16S sequencing. Studies were eligible if they provided publicly available paired-end raw sequencing data with corresponding BioProject accession numbers and sufficient metadata for downstream analyses. Raw FASTQ files were downloaded from the NCBI Sequence Read Archive (SRA) and reprocessed using a standardised QIIME2-based bioinformatics pipeline. Summary statistics for sequence preprocessing are provided in **Supplementary Table S1**. Samples with fewer than 1,000 total reads were excluded from downstream analyses. After quality filtering, feature tables, taxonomic assignments, and study-specific metadata were imported into phyloseq, and each dataset was retained as an individual phyloseq object for subsequent integrative analyses.

### Alpha, beta-diversity analysis

Alpha diversity was calculated on a per-study basis from phyloseq objects using functions implemented through the vegan package. The amplicon sequence variants (ASV) abundance tables were converted to numeric sample-by-feature matrices, and richness and diversity indices were estimated, including observed features, Chao1, and Shannon diversity. These indices were merged with selected metadata fields, including disease group and sampling site, and then compared between OSCC and control groups within each study. Group-wise visualisation was performed using violin plots with per-study statistical annotation. For cross-study beta-diversity analysis, taxa were agglomerated at the species level and converted to relative abundance profiles before distance calculation. To harmonise feature identities across datasets, species-level labels were reconstructed using combined genus–species identifiers, and unique sample names were generated by prefixing each sample with its study identifier. Bray–Curtis dissimilarity and principal coordinates analysis (PCoA) were then used to evaluate between-sample compositional variation across studies and sample types.

### Relative abundance profiling and biomarker heatmaps

To compare dominant taxa across studies, each phyloseq object was agglomerated to the genus level and transformed to relative abundance. Mean genus-level relative abundance was calculated within each disease group for each study, and the top-ranked genera were selected for visualisation. Relative abundance categories were binned into predefined intervals and displayed in dot plots, with point size representing abundance and colour indicating phylum-level taxonomy. For biomarker identification, taxa were collapsed at the genus and species levels and Linear discriminant analysis (LDA) Effect Size (LEfSe) analysis was performed. Significant taxa that were found in at least 3 studies were summarised across datasets, and study-wise directional changes between OSCC and Control groups were represented using LDA score heatmap.

### NetMoss2-based microbial network analysis

To compare microbial interaction patterns between OSCC and control groups, study-specific genus-level relative abundance tables were generated from phyloseq objects. Genus-level abundances were normalised using total-sum scaling, and genera present in more than 20% of samples within each study were retained to reduce the influence of sparsely observed taxa that may generate unstable or spurious correlations [45]. Filtered abundance matrices were then split into control and OSCC groups and exported for network construction. Microbial association networks were inferred using NetMoss2, with Sparse Correlations for Compositional data (SparCC) selected as the correlation method. Separate case and control networks were built from the exported abundance tables, and NetMoss scores were calculated by comparing the two group-specific networks. Genera with an adjusted *P* < 0.05 and a NetMoss score > 0.30 were retained as candidate network biomarkers. The resulting node-level NetMoss scores were ranked to identify taxa contributing most strongly to network rewiring between OSCC and Control groups. For visualisation, taxa were ordered by NetMoss score, and study-aggregated log2 fold change between case and control abundance was additionally calculated to label taxa as enriched in either group.

### PICRUSt analysis

Predicted functional profiles were analysed using KO abundance tables derived from PICRUSt output. ReporterScore analysis was then performed on the aligned KO tables. Differential-abundance *P* values for individual KOs were obtained using edgeR. These KO-level *P* values were matched back to the KO test table, adjusted using the Benjamini–Hochberg procedure, converted to directed Z-scores, and then aggregated into pathway-level reporter scores. Significant pathways were defined as those with absolute ReporterScore > 1.64 and adjusted *P* < 0.05. This threshold was applied both to individual study results and to the multi-study summary. For integrative comparison across studies, pathway-level results were collected, and pathways detected as significant in more than two studies were retained for recurrent-signal summarisation. Signed reporter score matrices were then constructed and visualised as heatmaps, with positive scores indicating enrichment in OSCC and negative scores indicating enrichment in controls.

### Statistical analysis and visualisation

All analyses were conducted in R. Community ecological analyses were performed using phyloseq and vegan; taxonomic visualisation used ggplot2, ggpubr, and pheatmap; differential functional profiling used pctax and ReporterScore; and network analysis used NetMoss2, igraph, and associated plotting/export routines. *P* values were adjusted using the Benjamini–Hochberg method where specified in the workflow.

## Supplementary Material

Supplementary Fig S1.png Supplementary Fig S1.png

Supplementary Fig S2.png Supplementary Fig S2.png

Supplementary Figure legends.docx Supplementary Figure legends.docx

Supplementary table S1.xlsx Supplementary table S1.xlsx
