# Supplementary material for: Integrative meta-analysis of the OSCC microbiome: from niche-specific heterogeneity to universal network and functional signatures
Source: J Oral Microbiol. 2026 Sep 7;18(1):2730638. doi: 10.1080/20002297.2026.2730638 (PMC13552301; doi:10.1080/20002297.2026.2730638)
Supplement: Supplementary file 1 [file ZJOM_A_2730638_SM2327.docx]

**Supplementary Figure legends**

**Figure S1. Sensitivity analysis of NetMoss results.** Leave-one-dataset-out and leave-two-datasets-out analyses were performed to evaluate the robustness of the NetMoss results.

**Figure S2. Stability of top-ranked NetMoss taxa across dataset-omission analyses.** The retention of top-ranked NetMoss taxa was evaluated across leave-one-dataset-out and leave-two-datasets-out analyses.
